# A systematic review of physical activity: benefits and needs for maintenance of quality of life among adults with intellectual disability

**DOI:** 10.3389/fspor.2023.1184946

**Published:** 2023-06-08

**Authors:** Udeme Samuel Jacob, Jace Pillay, Ensa Johnson, Oluwatomilayo (Tomi) Omoya, Adewale Philip Adedokun

**Affiliations:** ^1^ South African Research Chair: Education and Care in Childhood, Faculty of Education, University of Johannesburg, Johannesburg, South Africa; ^2^ Department of Inclusive Education, College of Education, University of South Africa, Pretoria, South Africa; ^3^ College of Nursing and Health Sciences, Flinders University, Adelaide, Australia; ^4^ Department of Special Education, Faculty of Education, University of Ibadan, Ibadan, Nigeria

**Keywords:** physical activity, interventions, intellectual disability, benefits, quality of life

## Abstract

The inactivity of people with intellectual disabilities (PwID) is a major contributor to ill health. Probably because people with intellectual disabilities are not adequately informed about physical activity and intervention programs required to enhance their fitness. This study critically reviewed physical activity: benefits and needs for maintenance of quality of life among adults with intellectual disability. An extensive search of bibliographic databases such as PubMed, PsycINFO, BioMed Central and Medline identified 735 academic papers. The research rigour was evaluated, and the validity of the findings was established. Based on the inclusion criteria, 15 studies were included in the review. Various forms of physical activity were studied as interventions. The results of a critical review indicate that physical activity has a moderate to strong positive impact on weight loss, sedentary behaviour, and disability-related quality of life. Adults with ID may benefit from physical activity as a non-pharmaceutical method of improving their health needs. However, this study's results may only apply to some adults with intellectual disabilities. The sample size needs to be increased in future studies in order to draw generalizable conclusions.

## Introduction

Intellectual disability (ID) is associated with considerable impairments in cognitive performance, resulting in the exhibition of maladaptive behaviour that originates before age 22 (1, 2). In addition to affecting the nervous and sensory systems, this disability may lead to metabolic and degenerative disorders, impaired ability to function, and physical disabilities (3). It significantly reduces the ability to comprehend, apply new and complex information. During the developmental period, intellectual and developmental disabilities (IDD) result from impairments in intellectual functioning and adaptive skills that adversely affect domains such as cognitive, functional, and social (4). Intellectual disability begins before adulthood, affects one's ability to function independently, and is detrimental to development on the long run (5). In the United Kingdom, intellectual disability is known as learning disabilities (6, 7), whereas in the United States of America, it is intellectual developmental disorder (7).

It is estimated that 10.37 people out of 1,000 live with this condition (8), affecting their ability to cope independently and affecting their development for the rest of their lives. Inequalities in health and social status are significant problems for many adults with ID (9). The evidence-based benefits of increased physical activity (PA) are also not enjoyed by a third of adults with ID (10). Health and social exclusion are aggravated by the lack of involvement in PA. Adults with ID face a global burden of health and social care costs (8).

Moreover, a person's inability to cope with social demands based on age can be categorized further according to severity (11). Apart from methodological differences between studies, estimates of ID prevalence vary by country (12), level of economic development, and individual characteristics (8). Some characteristics are associated with intellectual disabilities, including poor verbal skills, difficulty grasping the numerical concept, poor personal hygiene, such as using the bathroom, and emotional disorders or behavioural problems (13). Moreover, these individuals have difficulty balancing, separating, and connecting their movements. Their walking speed is noticeably slower the typical, thus restricting their physical or environmental movements, leading to little or no physical activities (14).

Inactive lifestyles or inability to engage in PA contribute significantly to high lifetime costs of health and non-healthcare services associated with diagnoses of ID. In addition to the economic costs associated with ID—or the resources used or lost because of the IDs—people with ID often require lifelong treatment and services (15). Cognitive impairments prevent persons with intellectual disability from actively participating in physical activities. Their appetites tend to be higher than those without intellectual disability, resulting in difficulties maintaining a healthy weight and an increased rate of obesity due to their appetite (13). These disabilities are more expensive to diagnose than other disabilities (such as cerebral palsy and visual and hearing impairments) (16). In some cases, they may be minimised by modifying one's lifestyle (12, 17).

In spite of the considerable health benefits of physical activity, it is believed that individuals with ID engage in relatively fewer PA, which makes it somewhat difficult for them to attain the expected level of health (10, 18). Lin et al., (19) suggested that people with ID should be physically active at least exercise 3 times per week and 30 min per, at a moderate intensity, for thirty minutes to improve their quality of life. The low levels of PA practice in this population have led to several articles focusing on identifying the factors that hinder PA practice in this population. Studies have identified a few factors that limit accessibility to PA practices, including transportation problems, cost, lack of personalized support and choices as well as lack of community PA programs (20–22). In the light of this reality, it is only logical that we should consider various ways PA can be of benefits to adults with ID. Relevant literature provides studies addressing this concern.

In terms of quality of life (QoL), adaptive behaviour is the most significant predictor (23). A person with IDD often has impaired executive function, which affects their ability to perform daily activities (24). Adults with ID have higher morbidity rates and shorter life expectancies (25, 26). As a result, individuals with ID are prone to age-related health problems at an earlier age, thus interventions that promote health status are beneficial for them (27). Most adults with ID do not strictly adhere to PA recommendations (28). Considering the above, promoting PA is essential for adults with ID.

The benefits taking part in PA includes enhances cardiorespiratory fitness as well as muscular strength. It contributes significantly to healthy weight loss (29). Another evidence showed that physical activity enhances performance and reduces the incidence of high blood pressure, weight gain, stroke, and blood sugar levels (13, 30). Adults with ID have a declining PA rate, which means they do not exercise enough to achieve optimal health. Sedentary behaviour resulting from poor physical activity is prevalent among adults with ID (25). Other findings have equally shown that adults with ID tend to experience fatigue-related diseases, low fitness levels, and obesity (25, 26) more than the general population health risks and mortality. PA is vital for healthy living because it reduces cardiovascular disease, hypertension, cancer, diabetes, and weight gain (31).

Every adult between 18 and 64 years old should exercise at least 150 min a week irrespective of their health status (32). Nevertheless, adults with ID have a relatively low prevalence of PA in this population subgroup. Individuals with ID participate in approximately 17.5%–33% of the recommended physical activity levels (33). The prevalence of obesity was higher among adults with ID than in adults without ID (34). Physical fitness and cardiovascular endurance are lower in people with ID than those without ID (35). Recognizing the health-promoting implications of PA on psychological well-being is imperative in determining the type, duration, frequency, and approach interventions that will improve quality of life (36). Based on the foregoing, this study was designed to assess and collate global research evidence on using physical activity among adults with ID. This study also examined gender differences in physical activity among adults with ID. Moreover, the review investigated the effect of physical activity on the experimental group. Hence, this study was guided by the following research questions:

1. What are the benefits of physical activity for adults with ID?
2. Is there a significant effect of physical activity on adults with ID based on gender difference?
3. Did physical activity significantly enhance the performance of adults with ID in the experimental group?

## Methodology

A search of PubMed, Scopus, PsychINFO, Cochrane library, and Ebsco databases was conducted using keywords and medical subject headings (MeSH). We used the following keywords in isolation and in combination*: “physical activity”, “Adults”, “intellectual disability”, “exercise”, “therapy”, “physical health”, “lifestyle factors”, and “Barriers and facilitators”.* Initially, no inclusion or exclusion criteria were applied to the literature search (see PRISMA flow chart below).

### Selection criteria

This systematic literature review aimed to conduct a **systematic review of physical activity: benefits and needs for maintenance of quality of life among adults with intellectual disability**. As explained in the section on data reduction, the search resulted in data sets that were then classified into predetermined categories. PRISMA established screening criteria (37). There was no limit to the location of the search. Vickers and Smith (38) found that excluding dissertations or including them did not affect the conclusion of the systematic literature review. All articles were screened independently based on the research objectives.

### Outcome of search

**Figure 1:**
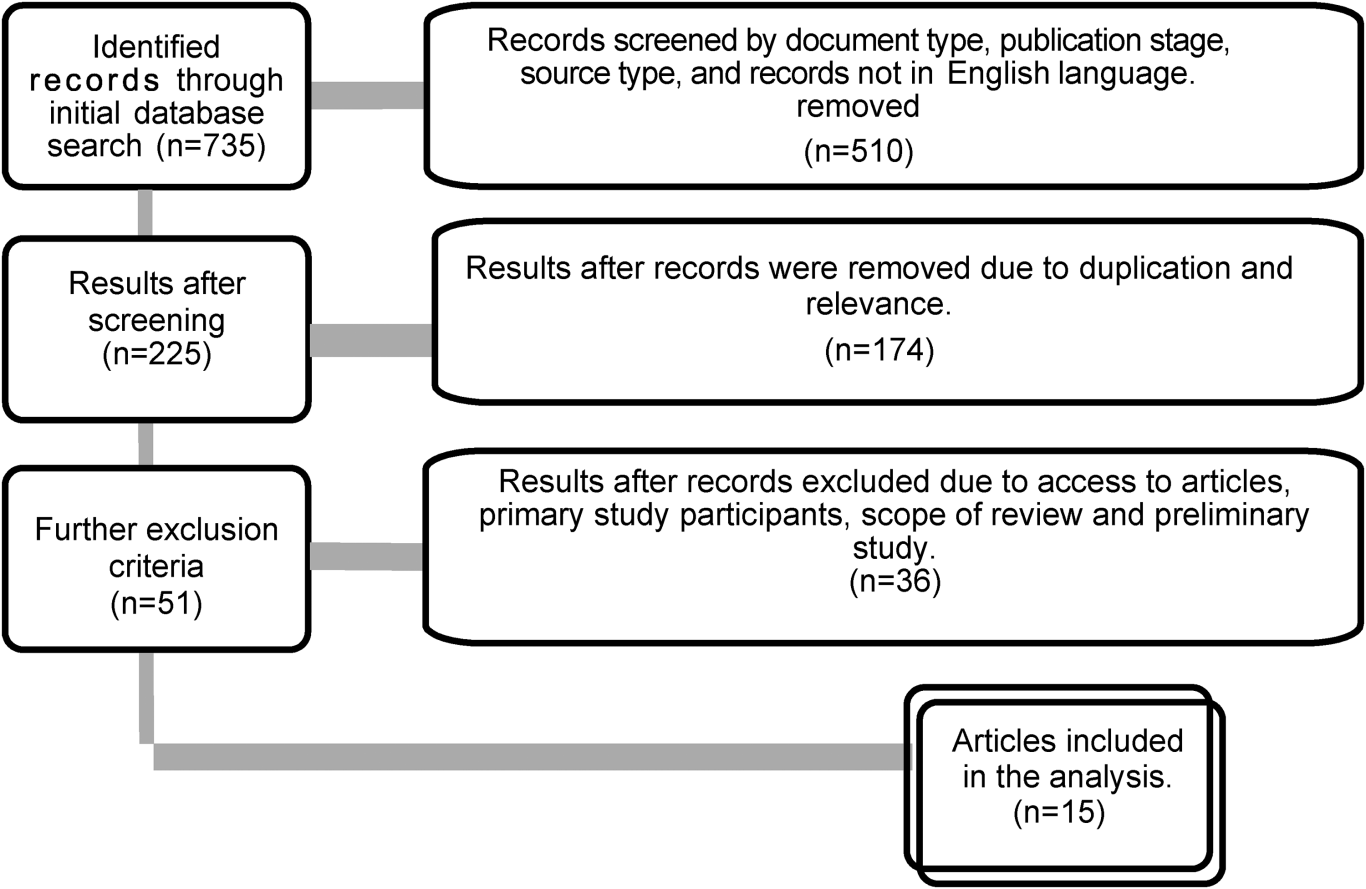
Flowchart of the systematic literature search.

Our search identified 735 publications (PubMed, Scopus, PsychINFO, Cochrane library and Ebsco) (see Figure 1). Before searching, we established criteria for including and excluding records. We removed 510 of the 225 articles due to their type, publication stage, source type, and publication language. We further screened the remaining 225 articles for duplication and relevance to the review, resulting in 51 articles being removed. Twenty-five of the remaining 51 articles were excluded based on access to articles, study participants, the scope of the review, and preliminary findings (39). A total of 15 studies were reviewed in full. The study included only original research articles.

### Data extraction

Two raters blind coded the 15 articles using a variety of demographic variables and effects of PA on people with ID (see Table 1 for details). In this study, some measures were coded, including the purpose, sample size (males and females), sampling technique, study design, instrument, and outcomes (pre and post). The information gathered was validated by two experts. All identified studies were evaluated and critiqued by two colleagues with higher degree status.

**Table 1:** Included studies, sample size, study objectives and major findings.

Author(S)	Sample Size	Study Design	Instrument	Duration of intervention	Gender (M/F)	Objectives	Major finding
Carbó-Carretéa et al. (40)	529 adults with ID	Accidental, non-randomized sampling	Personal Outcomes Scale—Spanish Adaptation. Support Needs and Strategies for Physical Activity Scale.	Not specified	M = 296 F = 233 A relative = 462 Designated professionals = 522.	The study examined the impact of perceptions of physical activity and individualized support on each of eight quality-of-life-related domains and three higher-order quality-of-life factors.	The findings confirm that perception plays a relevant role in predicting the PA level of a person with ID. The results suggest that identifying support needs and providing adequate strategies in PA has an impact on enhanced personal outcomes.
Harris et al. (41)	66 adults with ID and obesity	Randomised controlled trial design.	Actigraph GT3X + accelerometers	12 months	Not specified	This study conducted a single-blind, cluster randomised controlled trial comparing a multi-component weight management programme to a health education programme.	Adapting an energy deficit diet to meet the needs of adults with ID and obesity was found to be a feasible and acceptable way to lose weight.
Kim & Yi (13)	17 Adults with ID	Not specified	Actical was worn for five days on workdays by 17 adults with ID to assess physical activity and metabolic syndrome risk factors.		M = 13 F = 4	The study investigated the relationship between PA and metabolic syndrome risk factors in adults with ID.	Muscle strength, muscular endurance, low-density lipoprotein cholesterol, and disability rating were positively correlated with PA among participants. A significant difference was found between adults with ID and those of similar age groups regarding PA.
Melville et al. (42)	54 adults with and without ID.	An open pilot study	Primary outcomes change in weight. Change in Body Mass Index Associations between patient characteristics and a weight loss	24 weeks	M = 22 F = 32	The present study examined the effectiveness of the TAKE 5 multi-component weight-loss intervention.	There was a significant decrease in body weight, BMI, waist circumference and daily sedentary behaviour among adults with ID and obesity. TAKE 5 proved to be a successful weight-loss intervention for adults with intellectual disabilities and obesity, according to the study.
Diz et al. (43)	16 adults with and without ID.	Not specified	An adaptation to a person's daily routine is assessed on the validated Portuguese version of the Adaptive Behaviour Scale (PABS).	20-weeks program included biweekly sessions of 50 min each, in a total of 29 sessions	M = 7 F = 9	The effects of a physical activity program on adaptive behaviour, motor proficiency, fitness, and quality of life of 16 adults with intellectual disability (ID) were analysed.	During baseline assessments, the control group scored higher, but after the program, the experimental group had higher average scores. The control group stabilized performance over time, and the experimental group improved in most domains, such as socialization, responsibility, and arm curls. Social inclusion did not present significant differences in the control group, but in the experimental group, there was an improvement, although not significant, over time.
Jo et al. (44)	12 adults with ID	Experimental study	In addition to cardiopulmonary endurance testing, bioelectrical impedance analysis, flexibility assessments, muscle endurance assessments, and strength assessments, measurement of health-related physical fitness included hand grip strength (hand grip strength). The physical self-efficacy scale was used to measure self-efficacy. Physical activity levels were measured using accelerometers.	90 min, twice per week for 12 weeks	Not specified	An exercise program was evaluated for its effect on self-efficacy and physical activity levels among adults with ID.	The experimental group's exercise levels, self-efficacy, and muscle endurance significantly improved after the intervention.
Font-Farré et al. (45)	48 adults with and without ID.	cross-sectional study,	The 6MWT is used to assess the submaximal exercise capacity of individuals	Not specified	Not specified	The study aimed to describe and compare cardiac autonomic modulation before, during, and after the six-minute walk test (6MWT) in older adults with and without ID, in a group of older adults with and without ID.	Participants without ID showed significantly better results in heart rate kinetics and time variables during recovery. Recovery HR kinetics after the 6MWT was slower in older adults with ID
Oviedo et al. (46)	84 participants	cross-sectional study,	Weight and height were used to compute Body Mass Index (BMI). The percentages of body fat and fat-free mass were also determined. GT3X Actigraph accelerometers were used to measure PA levels and sedentary time patterns.	Not specified	M = 49 F = 35	The study analyzed sedentary time and PA patterns among adults and older adults with ID.	Adults performed higher amounts of total PA and moderate to vigorous PA than older adults during the week, on weekdays and in center time. No differences between males and females were found for either PA levels or ST.
Chow et al. (47)	114	cross-sectional study,	A physical fitness test is conducted (percent body fat, waist circumference, 6-minute walk, arm curls, and sit and reach).	Not specified	M = 71 F = 43	Adults with mild to moderate ID who lived in four group homes or worked in sheltered workshops were examined for their habitual daily PA and health-related physical fitness (PF).	No significant differences between mild and moderate ID were found for any PA or physical fitness variable. Linear multiple regression analyses showed 6MWT to be the only significant PF variable contributing to the variance of PA and sedentary behavior. In conclusion, adults with ID reside in group home have low PA and low fitness levels.
Oviedo et al. (48)	92 adults with mild to moderate ID	cross-sectional design	Body Mass Index (BMI). Physical activity levels and Sedentary Time were assessed with GT3X Actigraph accelerometers	7 days	M = 51 F = 41	This study describes and compares physical activity (PA) levels and sedentary time (ST) of active (AG) and a non-active (NAG) group of adults with intellectual disability (ID) vs. a group of adults without ID.	The participants of the active group did not demonstrate less ST than the non-active group. The results indicate that well-designed and integrated PA programs may increase PA levels in the population with ID.
Hsu et al. (49)	60 individuals with ID	cross-sectional study	The ActiGraph GT3X monitor was used to measure physical activity for seven days. In order to evaluate physical fitness, a 6-minute walk test was administered, as well as isometric push-ups, modified curl-ups, handgrip strength tests, and back-saver sit-and-reach tests.	7 days of PA	M = 33 F = 27.	This cross-sectional study assessed the associations of gender, age, level of intellectual disabilities (IDs) and of daily sedentary and physical activity (PA) time with physical fitness in adults with ID.	Age and gender were associated with lower performance in multiple aspects of physical fitness among women aged 39 and older. Moderate-to-vigorous PA (MVPA) was linked with enhanced muscular strength and endurance.
Tyrer et al. (50)	920 adults with ID	cross-sectional analysis	Characteristics of the participants Multimorbidity Common risk factors	Not specified	Not specified	The study aimed to determine the prevalence of multimorbidity in a population of adults with ID. The study also aimed to identify this population's risk factors, including lifestyle factors, for multimorbidity.	The prevalence of multimorbidity was 61.2% Individuals who were physically inactive or sedentary were more likely to be multimorbid, independent of ability to walk, age, gender, severity of ID, ethnicity, and socio-economic status
Olsen et al. (51)	214 adults with ID	Community based cross-sectional survey.	The POMONA-15 health indicators were used.	Not specified	Not specified	The study investigated the associations between perceived health and demographics, degree of ID, physical health conditions, and weight and physical activity level. The study also examined lifestyle factors and multimorbidity as predictors of perceived health adjusted for age, gender, and level of ID.	There were significant associations between poor health ratings and female gender, lower motor function, number of physical health conditions and several indicators of PA levels. A lack of physical activity tends to negatively influence perceived health.
Zwack et al. (52)	68 young adults with ID	Cohort study	Clinical assessment physical assessment Cardiovascular and metabolic measurements Food Choice Questionnaire (modified Dietary Quality Tools)	Not specified	Not specified	The study investigated the relationship between modifiable risk factors and cardiometabolic health profiles. It evaluated traditional cardiometabolic parameters, physical activity levels, diet, and associated health knowledge among adults with ID aged 18–45 years.	Knowledge about nutrition and physical activity appears to be an important predictor of cardiometabolic risk in this population
Mitchell et al. (53)	19 adults with ID	Qualitative research	Semi-structured interviews or focus groups	Not specified	Not specified	The study investigated the perceived benefits, barriers, choices, and use of intervention resources.	There were four overarching themes: perceived benefits, perceived drawbacks or barriers, walking choices, and using Walk Well resources. Carers also played an important role in facilitating and preventing behaviour change in adults with intellectual disabilities.

### Description of participants

Table 1 revealed that the included studies were conducted between 2011 and 2022. There were two thousand three hundred and three (2,303) participants in the fifteen (15) studies reviewed. There were 920 participants in the study conducted by Tyrer et al. (50), representing 38.9% of the study participants, which is the study with the highest number of participants. There were 529 respondents in Carbo-Carretéa et al. (40), representing 22.4% of the study participants included in the review. Diz et al. (43) had the least number of participants that is 16 (0.67%), while Kim and Yi (13) had 17 (0.72%) participants. Each other study included less than 1% of the total participants.

The table also showed that the primary participants in 10 (63%) studies were described as adults with ID, while 1 (6%) study participants were young adults with ID. Moreover, four studies used mixed participants. One study (6%) involved adults with ID, their professional service providers, and relatives, while three (19%) involved adults with and without ID. 1 (6%) study had participants with comorbid conditions (adults with intellectual disabilities and obesity).

### Study objective

The objectives of the studies included in this review varied significantly. Carbo-Carretéa et al. (40) investigated how perceptions of PA affect the quality of life using eight categories and three higher-level quality-of-life factors. A single-blind, cluster-randomised controlled trial compared a multi-component health education programme to a weight management program (41). Researchers also investigated the impact of physical activities on metabolic syndrome risk factors (13), quality of life, motor proficiency, and fitness levels (43) and fitness levels, self-efficacy, and activity levels (43) among adults with ID.

Melville et al. (42) evaluated the efficacy of TAKE 5, an intervention designed to promote weight loss. The objective of Chow et al. (47) and Oviedo et al. (48) examined the relationship between PA levels and sedentary time, while Hsu et al. (49) examined the correlation between physical fitness and gender, age, degree of ID and sedentary time and PA time. Tyrer et al. (50) and Olsen et al. (51) examined risk factors for multimorbidity, including lifestyle factors. Olsen et al. (51) also considered the relationship between perceptions of health and demographics, degree of ID, weight and PA, and overall health condition. Zwack et al. (52) examined the relationship between reversible risk factors and cardiometabolic profiles. Mitchell et al. (53) examined participants' perceptions of benefits, barriers, and choices in a walking program as part of a study.

### Research design

Additionally, the table provides information on the research design used in the studies included in this review. In the review, 7 (43.8%) studies employed a cross-sectional research design (45–51). There are also several other research designs used in the included studies, including accidental, non-randomized sampling (40), randomized controlled studies (41), an open pilot study (42), an experimental study (44), and qualitative studies. It should be noted, however, that 3 (18.8%) of the studies did not specify the research design employed.

### Participants gender

Table 1 also showed that 8 (50%) of the included studies indicated the gender of participants while the remaining 8 (50%) did not. 542 (23%) of the participants were identified as male while 424 (18%) were identified as female. The gender of 1,399 (59%) participants was not specified. The number of male participants were more than female by 118. A study had more female that is 32 compare male 22 (42) while another study had the lowest number of female participants that is 4 to male that is 13 (13). It is worth noting that the table also revealed the sampling technique for selection of study participants in the included study and the instrument used for data collection. Two studies collected data on body index mass of participants **(**42, 46, 48). A study employed semi-structured interviews or focus groups for data collection (53). Self-efficacy scale was used in one study (44). A scale for assessing physical activity known as accelerometers was used in three studies (44, 46, 48). In one study physical activity was monitored for 7 days using an ActiGraph GT3X monitor (49).

### Sampling technique

Five studies used convenient sampling technique for the selection of participants in the study (43, 45–47, 53). Purposive sampling technique was adopted in three studies for participants selection (44, 48, 50). However, 5(38%) of the included studies describe the procedure for sampling but did not specify the sampling technique (13, 40–42).

## Culminative main findings

### Benefits of physical activity

#### Improved quality of life

The results revealed high values for PA, especially for the well-being factor (Carbo-Carretéa et al. (40) and the relationship between fitness and health was significant (44). The correlation between physical activity and muscular strength, endurance, total cholesterol, low-density lipoprotein cholesterol, and disability rating was confirmed (13). Despite being not statistically significant, Diz et al. (43) found that the PA program appears to have a moderate positive impact on QOL: experimental group participants reported better scores in both versions. Font-Farré (45) results showed that differences in heart rate variability variables between groups were insignificant. According to Hsu et al. (49) flexibility and endurance were associated with more light-intensity PA (LPA).

#### Weight loss

In persons with ID, disability ratings, glycated haemoglobin, and physical activity levels were correlated by 73.9% (13). Subjects' demographic variables and activity levels differed significantly (P0.001), but not their age or body mass index. The exploratory efficacy analysis by Harris et al. (41) revealed that at 12 months, nearly five times as many participants in TAKE 5 (50·0%) achieved 5%–10% weight loss, compared to Waist Winners Too (20·8%). Adults with ID and obesity can benefit from an energy deficit diet incorporated into a multi-component programme for weight loss. Melville et al. (42) concluded that weight (mean difference) decreased significantly (−4·47), body mass index (−1·82) and waist circumference (−6·29) where *P* < 0·0001 and participants' daily sedentary behaviour (−41·40) where *P* = 0·00034. In addition, thirteen participants (36%) lost at least 5% of their original body weight after the intervention. Diz et al. (43) showed that physical activity improved balance and strength. However, the improvement could have been more significant, allowing for enhanced performance in everyday activities like sitting and walking.

#### Sedentary life

Oviedo et al. (48) reported that the study provides a more comprehensive understanding of PA levels and sedimentary time in groups of active and inactive adults with and without ID. It is consistent with other studies that, regardless of gender or age, adults with ID are more sedentary and perform less light PA (54, 55). Oviedo et al. (46)) found that adults with ID were more physically active than older adults with ID regarding moderate-to-vigorous PA, but there was no difference in ST. The influence of moderate to vigorous PA on total PA was similar in previous studies with the general population and with individuals with ID (56, 57).

A significant difference was not observed in physical activity, sedentary behaviour, and physical fitness variables between non-overweight/nonobese (BMI < 23) or obese (BMI ≥ 23) people, nor was there any difference between those at low and high risks for central obesity (47). Furthermore, Oviedo et al. (48) found that participants with ID tended to engage in more bouts of sedentary behaviour per day, regardless of gender or age, compared to participants without ID. Some underlining factors may contribute to this. Depending on the centre, participants with ID will take similar scheduled breaks, such as tea and lunchtime. It is also important to note that adults with ID have less opportunity to participate in PA after work.

The likelihood of multimorbidity increased with physical inactivity or sedentary behaviour, regardless of age, gender, the severity of ID, ethnicity, or socioeconomic status (50). Researchers found that sedentary behaviour, not physical activity, still negatively affected health (50) even when lifelong conditions were excluded. Additionally, this population's nutrition and physical activity knowledge appear to significantly predict cardiometabolic risk (52). Oviedo et al. (46) found that light PA values were similar to those reported by Philips & Holland (57) but higher than those found by Melville et al. (42). The difference with Melville et al. (42) stems from the fact that the participants in that study were four years older with a BMI ≥ 30 kg/m2.

#### Effect on physical activity based on gender difference

Oviedo et al. (48) revealed that females from the group without intellectual disability engaged in more physical activity than females from the non-activity group with ID. As a result of the lack of participation in physical activity programs and the low-intensity tasks conducted at centres, the total physical activity of females with ID was lower. Physical fitness variables, except for body fat percentage, did not differ by sex during a 6-minute walk, arm curl, and sit-and-reach test (47). However, Hsu et al. (49) reported that female gender above 39 years old were associated with lower performance in multiple aspects of physical fitness.

According to Oviedo et al. (46), males and females did not have different PA levels or ST. Based on these results, older males and females in Oviedo et al. (46) should engage in activities specific to their age and gender to increase their PA levels. In univariate analyses, the number of PA conditions, lower motor function, and multiple physical activity indicators correlated with poor health ratings (51). Oviedo et al. (46) found that females in the non-ID group performed higher amounts of total PA than those in the non-active group.

There was a significant difference between male and female participants regarding abdominal muscular endurance and grip strength; these results are consistent with research suggesting that men with ID have higher muscular strength and endurance (58). It has been found, however, that women with ID have less cardiorespiratory endurance (59) and greater flexibility (58). Oviedo et al. (46) found no statistically significant differences between genders for the parameters studied. This supported the findings of Gawlik et al. (59) that aerobic capacity calculated per kilogram of body weight did not differ by gender.

#### Effect of physical activity on experimental group

The experimental group significantly enhanced muscle endurance, self-efficacy, and levels of physical activity (43). Diz et al. (43) observed that controls impair children's physical development, responsibility, and socialisation and negatively affect their interpersonal interactions. A significant difference was found between participants in the experimental group, although effect sizes tended to be moderate, indicating better performance following the program.

Diz et al. (43) reported that after the program, the experimental group participants reported higher levels of satisfaction, despite the higher levels of social inclusion, individual autonomy, intellectual development, and general well-being reported in the control group initially and in the self-report version. The proxy version assumes greater values only for the control group's interpersonal relationships and emotional well-being domains. Neither age nor body mass index was significantly correlated with differences in demographic variables or activity levels (13).

## Discussion of finding

This systematic review evaluates the physical activity benefits and needs of adults with ID. The predetermined inclusion criteria were met by 15 of the 1,005 studies identified as potentially relevant. This review has identified various ways physical activity can be of benefit and use to ensure an improved quality of life among persons with ID. The use of physical activity as an intervention group varied significantly between the studies in terms of content, structure, and duration. The studies did not use a single outcome measure. In the studies, intellectual disability was referred to using relatively consistent terminology. Three studies used a purposive sampling technique, while five used a convenient sampling.

The results from the meta-analysis show variation in the effect of PA on quality of life of adults with ID. The prevalence of factors related to well-being and fitness was high (40, 44). It is partially consistent with the results of Calders et al. (60), which showed that exercise improved muscle endurance, strength, and cardiovascular endurance in adults with ID after 20 weeks. Jo et al. (44) reported significantly different results from Wu et al. (61). The exercise intervention in Jo et al. (44) was shorter and less intense than Wu et al. (61), a 6-month exercise intervention. There may also be a difference because previous studies used different measurement tools.

Several factors make it difficult to generalize the participants' PA levels to the general population of adults with ID. This review's data were derived from a selective sample, which doesn't seem to represent the ID community, so their activity levels were likely higher than those of the general ID population. The study design and instruments for data collection varied significantly. Additionally, the duration of the included studies varied widely from as low as 7 days to as long as 12 months. Although, the study by Carbo-Carretéa et al. (40) confirmed that physical activity perceptions and individualized support habits are associated with improved quality of life.

Diz et al. (43) concluded that controls showed a better adaptive profile in most adaptive domains, except for time and numbers, sexual behaviour, and social interaction. This group's scores remained the same over time, while the experimental group's performance improved in most areas. As a result, Kim and Yi (13) advocated for more research on improving and enhancing physical activities in sheltered workshops, where adults with ID receive the most supervision and guidelines for maintaining their health. Physical activity and fitness variables did not differ significantly between mild and moderate ID (47). Carbó-Carretéa et al. (40) show that identifying support needs and providing appropriate strategies in PA is associated with enhanced outcomes for individuals.

The benefits of physical activity include improving strength and reducing the prevalence of chronic diseases, including hypertension, obesity, stroke, and diabetes (30). Physical activity improves strength and reduces chronic diseases such as hypertension, obesity, strokes, and diabetes (30). The difficulty of carrying out daily activities, avoiding movement, and becoming physically inactive is common among individuals with disabilities. Since they cannot participate in the same amount of physical activity as adults without disabilities, their weight and metabolic syndrome frequencies increase (62). If this inactivity continues into adulthood, metabolic syndrome and related chronic diseases will likely develop (13).

It was found that the improvements continued a month after the program ended despite a slight decline in retention assessments (43). Despite this, there were significant improvements in effort items, concentration, and agility. There were four overarching themes identified by Mitchell et al. (53): perceived benefits of participating in the programme, perceived disadvantages, walking options, and resources for walking. Participants reported positive experiences with the programme, despite no significant increase in walking. An integrated weight management programme for adults with intellectual disabilities can be tested in a full-scale trial (41). Researchers recruited adults with all levels of intellectual disabilities using the multi-point recruitment strategy, which overcame barriers to recruitment for adults with ID.

There were no statistically significant differences in testing the homogeneity between the experimental and control groups (44). Consequently, the researchers concluded that there were no initial differences in the data. Font-Farré et al. (45) concluded that differences between groups in heart rate variability were not significant. Results showed that participants without ID had significantly better heart rate kinetics during recovery than those with ID.

## Conclusion

This study reviewed PA therapies delivered by different specialists for adults with ID. Further research is needed to determine whether PA programs lead to positive outcomes among adults with ID. The findings of our study cannot be directly compared to those of existing studies. Promoting regular PA engagement among adults with ID should be put forward on national and international agenda and considered non-pharmacological support for a more active life and a healthier lifestyle. As a result of reviewing fifteen studies, the authors found that the treatment had varied significant effects on individuals with intellectual disabilities, including weight loss, sedentary behaviour, and improved quality of life for individuals with intellectual disabilities. Based on physical activity, tests of abdominal muscular endurance and grip strength were also statistically different between males and females.

The findings highlight the importance of actively participating in research and considering the complexities of PA for individuals with ID. In addition, caregivers, stakeholders, and other community members must be informed about PA's benefits for adults with ID. A larger sample size is necessary to establish a generalizable conclusion for future research. Based on an existing review, there is evidence that interventions for individuals with ID are effective. Consequently, our review complements the findings of other reviews and is highly relevant to the needs and benefits of physical activity for adults with ID. Our review has several limitations, which should be noted. We only included open-access publications due to resource limitations. Secondly, we used a reductionist approach, excluding articles that were not about PA interventions for adults with ID.

Additionally, a publication bias remains since we excluded publications in journals and books (excluding studies that are in press). This review did not allow the analysis of possible mediating and moderating factors that may contribute to barriers to PA for adults with ID because of maximizing study homogeneity.
